# Integrated care to address child and adolescent health in the 21st century: A clinical review

**DOI:** 10.1002/jcv2.12045

**Published:** 2021-10-23

**Authors:** Mina Fazel, Alice Townsend, Harriet Stewart, Maryland Pao, Isabel Paz, Jane Walker, Susan M. Sawyer, Michael Sharpe

**Affiliations:** ^1^ Department of Psychiatry University of Oxford Oxford UK; ^2^ The Oxford Psychological Medicine Centre Oxford University Hospitals NHSFT Oxford UK; ^3^ Sunderland and South Tyneside NHS Foundation Trust Tyne and Wear UK; ^4^ Department of Health and Human Services National Institute of Mental Health National Institutes of Health Bethesda Maryland USA; ^5^ Centre for Adolescent Health Royal Children’s Hospital Parkville Victoria Australia; ^6^ Murdoch Children’s Research Institute Parkville Victoria Australia; ^7^ Department of Paediatrics The University of Melbourne Parkville Victoria Australia

**Keywords:** adolescent, child, collaborative care, disabilities, healthcare, integration, mental health, quality

## Abstract

**Background:**

Increasing specialisation and technical sophistication of medical tools across the 21st century have contributed to dramatic improvements in the life‐expectancy of children and adolescents with complex physical health problems. Concurrently, there is growing appreciation within the community of the extent that children and adolescents experience mental disorders, which are more prevalent in those with complex chronic, serious or life‐limiting health conditions. In this context, there are compelling reasons for paediatric services to move to a model of care that promotes greater integration of child psychiatry within the medical, somatic teams that care for children and adolescents in children’s hospitals.

**Aims:**

In this article, we discuss the range of medical disorders managed by contemporary paediatrics.

**Materials and Methods:**

We conducted a broad review of the literature and existing services, and use individual accounts to illustrate adolescents’ healthcare preferences in the context of the challenges they experience around their mental health.

**Results:**

Relevant disorders include life‐limiting disorders, such as cancer; disorders involving the brain, such as epilepsy; common chronic disorders, such as asthma and diabetes; psychiatric emergencies, such as deliberate self‐harm; and conditions that most commonly present to paediatric services, but where psychiatric input is required, such as severe eating disorders, somatic symptom disorders and gender dysphoria. The persisting legacy of the historical separation of physical and mental health services is described. Yet there are many models of service integration that can promote more collaborative care between psychiatrists and medical specialists, including some which have been taken to scale.

**Discussion:**

In essence, clinical teams in children’s hospitals require more collaborative approaches that facilitate early recognition and treatment of the psychological aspects of illness as an integral part of patient‐centred, family‐focussed paediatric care, rather than as something that is bolted on when things go wrong.

**Conclusion:**

Whilst trust and goodwill between services and providers will be required for novel models of care to be implemented, evaluation of these new models and incorporation of young people’s healthcare preferences is needed.

## Graphical abstract

Increasing specialisation and technical sophistication of medical tools across the 21st century have contributed to dramatic improvements in the life‐expectancy of children and adolescents with complex physical health problems. Concurrently, there is growing appreciation within the community of the extent that children and adolescents experience mental disorders, which are more prevalent in those with complex chronic, serious or life‐limiting health conditions. In this context, there are compelling reasons for paediatric services to move to a model of care that promotes greater integration of child psychiatry within the medical teams that care for children and adolescents in children’s hospitals.

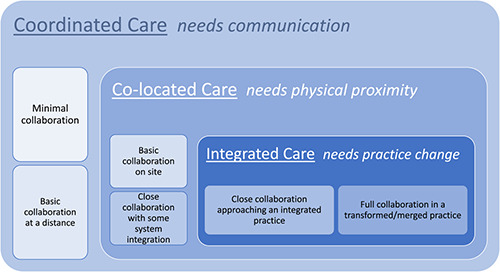

**Key points**

- As the science of medicine improves, children and adolescents with increasingly complex needs are being managed by paediatric teams.
- Better integration of somatic and psychological care is needed with models of collaborative care in children’s hospital settings being developed.
- The range of disorders that benefit from integrated care include cancer care, disorders involving the brain, common chronic conditions, psychiatric emergencies and chronic pain and eating disorder treatment.
- Incorporating the preferences of children and adolescents, as well as their families, into these models of care remains important.

## THE CHANGING FACE OF MEDICINE

There should be full collaboration between mental health and physical health services, and they should all be in the same place. [15 year old outpatient at a children’s hospital with somatic syndrome disorder]

The practice of medicine is continuing to evolve to meet changing health needs and community expectations. Alongside the changing cultural milieu in which today’s young people are growing up, there is a need to reconceptualise how health services assist children and families to manage chronic and increasingly complex health problems. Infancy, childhood and adolescence are each associated with developmental challenges that reflect dramatic development in physical growth, cognitive capacities, social relationships and communication abilities, all within a relatively short period of time. Over the past few decades, equally dramatic medical and technical advances have improved the survival of children and adolescents with previously fatal conditions. Many of these children are now growing up with a relatively heavy burden of healthcare, amid growing awareness by health care professionals of the important psychological, social and ecological contexts of care.

In high‐income countries, the complexity of needs of many patients in tertiary children’s hospitals reflects these advances and challenges, and raises questions about the extent to which current models of care are fit for purpose (Sharpe & Naylor, 2016). Previously the majority of paediatric patients would be managed by a single specialist, who could address the majority of their health care needs. In contrast, contemporary paediatrics is accompanied by a level of specialisation and sophistication of technical tools that commonly requires expert multidisciplinary teams (MDT) to manage complex chronic health conditions and disabilities. This shift, from a more singular orientation led by a key clinician towards more team‐based care for children and adolescents, forces us to reconsider not only how care is delivered, but by whom.

An additional challenge is the importance of responding to children’s and adolescents’ emotional distress. There is growing awareness of the extent of mental health difficulties in young people in general, especially anxiety and depression, as well as the value of identifying individuals and families who are most negatively affected by the social and environmental determinants of health. There is also growing evidence that children and adolescents with complex chronic health conditions and disabilities experience a higher prevalence of mental illness, which in turn impacts their somatic health outcomes.

In this clinical review, we discuss the value of greater integration of mental and physical health care for children and adolescents managed by tertiary children’s hospitals. To do this, we will describe clinical conditions that require a more integrated model of care where ‘all parts are brought together’ to work seamlessly for patients and their families (Royal College of Psychiatrists, 2019). We will explain some aspects of the history of the separation of paediatric and psychiatric services, briefly highlight some different models of integration of care and conclude with a call for greater integration to embrace the needs and demands of the current generation of younger patients and their families. Throughout this review, we include the voices of adolescents who are currently receiving care at a tertiary children’s hospital in the United Kingdom. We will use the term ‘child and adolescent’ to describe the population commonly seen in children’s hospitals, predominantly up to the age of 18 years.

## THE NEED FOR PSYCHIATRY IN CHILDREN’S HOSPITALS

The nature of services provided by contemporary children’s hospitals must reflect the breadth of clinical presentations that they manage. Evidence increasingly highlights the high prevalence of psychiatric and neurodevelopmental disorders in the general population of children and adolescents, and particularly in those with complex and chronic medical disorders (Sadler et al., 2018). This is especially the case for anxiety and depression, although behavioural problems and eating disorders are also notable. There are five main areas of activity that are particularly relevant to psychiatry in a children’s hospital:

- life‐limiting illnesses, such as cancer;
- disorders involving the brain, such as epilepsy;
- common chronic physical disorders, such as diabetes;
- psychiatric emergencies, such as presentations of self‐harm;
- disorders that commonly present to paediatric services but where psychiatric input is often required, such as severe eating disorders, somatic symptom disorders and gender dysphoria.

**FIGURE 1:**
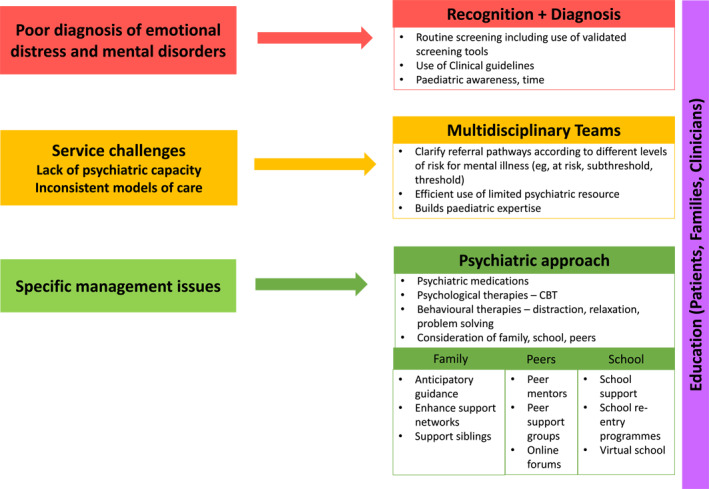
Areas of need for psychiatry services in Children’s Hospitals, with potential responses

Each of these areas will be briefly discussed, appreciating that some conditions span overlapping areas. This discussion has been informed by a narrative review of the literature as well as the views and experiences of young patients managed within an integrated clinical service in the United Kingdom. A summary of these data is presented in Appendix S1, whilst management approaches to consider are presented in Figure 1. Although the focus of this article is limited to child psychiatry and paediatric medicine, it is important to keep in mind that there are many different health care professionals that work within tertiary care settings with broad and wide‐reaching roles essential to both the psychological and physical care of the patients and families seen.

### Life‐limiting illnesses

Definitely having mental health support is very important for children with cancer. It is a massive thing and it affects you in every way, physically, emotionally and socially. You need someone there and then, you can’t wait. Having mental health support was very important for me, because I was very depressed. It was difficult for me, because I knew my life was in danger and having someone that understands and gives you the support that you need is invaluable. It also helped that I could tell them medical things I was struggling with, but was uncomfortable to tell the oncologist. However, sometimes I wished the support had been offered away from the hospital and the medical ward, as I was reminded constantly of the treatment and the threat of the illness. Ideally patients should choose whether they want to be seen in the same place or not. It is important that the people providing the mental health support know about the physical side of things, so that you don’t have to explain it over and over again. [18 year old patient with an acute leukaemia]

Conditions with a high disease burden, such as cancer, have been shown to increase the risk of psychiatric disorder (Kurtz & Abrams, 2011; Temming & Jenney, 2010) with importance placed on conceptualising psychological outcomes (Sansom‐Daly & Wakefield, 2013). For example, a study of 1345 adolescent survivors of leukaemia found an approximately 1.5 times increased risk of depression, anxiety, attention deficit and antisocial behaviours compared to siblings (Temming & Jenney, 2010). A further review of 5320 paediatric brain tumour survivors reported increased incidence of depression, anxiety, schizophrenia and related psychoses and behavioural problems compared to the general population (Shah et al., 2015).

The nature of the patient journey for a particular cancer is a predictor of its potential psychological effects. The care pathway from diagnosis of cancer to long‐term follow‐up usually spans a period of many years and involves repeated investigations, procedures, admissions and treatments. These experiences can have far‐reaching psychosocial repercussions, which have encouraged some countries to publish standards for psychosocial care (Wiener et al., 2015). For example, anticipatory anxiety is a common issue in clinical practice benefiting from a MDT approach involving judicious use of symptom‐specific medication, such as analgesia and anti‐emetics and psychological support, such as distraction and relaxation techniques (Kurtz & Abrams, 2011). The medical interventions required to manage cancer can disrupt engagement with social activities, school, peers, family, as well as the individual’s development of body image, identity and sexuality (Kurtz & Abrams, 2011). With improvements in prognosis, a growing cohort of patients transition from being a ‘cancer patient’ to a ‘cancer survivor’. This change in identity can be difficult to adjust to psychologically, which may not be reflected in the level and nature of support available as these adolescents will often then transition from hospital to community services (Kurtz & Abrams, 2011; Sawyer et al., 2017). The Childhood Cancer Survivorship Study of 20,276 patients reported a significantly increased risk of psychosocial issues compared to controls (Kurtz & Abrams, 2011; Wakefield et al., 2010). There are particular risks associated with different cancers and treatment type, but central nervous system (CNS) tumours, neurosurgery, chemotherapeutic agents and cranial radiation can all result in neurocognitive sequelae, further placing young people at particular risk for psychiatric comorbidities.

### Disorders involving the brain

When I first came to the hospital I thought my problems were physical. When I was told I didn’t have epilepsy I had a mixed bunch of emotions. Initially I was very confused because I had been taking antiepileptic medication for 5 years. It was hard for me to trust medical professionals. Now I really understand what epilepsy is, and that I don’t have it, but I needed help to understand it. If at that point I had been told I needed to go somewhere else to get mental health support, I would have felt that they were brushing me off. I would have been angry and annoyed towards the health care system. I probably would have become depressed. I don’t think I would have been able to trust medical professionals again [15 year old male presenting with seizures. His mother’s account is included in Appendix S2]

In addition to CNS tumours, neurological disorders, such as epilepsy and traumatic brain injuries (TBIs), are associated with a high prevalence of psychiatric comorbidities. This significantly increases the complexity of patient management, which is often not adequately reflected in best practice guidelines, highlighting the need for collaboration between psychiatry and neurology (Plevin & Smith, 2019, Salpekar et al., 2015). A recent review of children post‐TBI found the prevalence of depression was 33%–50% (Ryttersgaard et al., 2020). Novel psychiatric diagnoses post‐TBI include a spectrum of personality change, ADHD and other behavioural disorders (Max, 2014; Ryttersgaard et al., 2020).

A review of children with epilepsy (CWE) reported that psychopathology occurs in 37%–77% including depression, anxiety, intellectual impairments, ADHD and autism spectrum disorders (ASD; Verrotti et al., 2014). Another review reported that the risk of depression remains two to five times higher than the general population even when epilepsy is adequately controlled (Salpekar et al., 2015). The relationship between psychiatric symptoms and epilepsy is complex. For example, ‘stress’ is one of the most commonly self‐reported precipitants of seizures (Verrotti et al., 2014). The diagnosis of psychiatric disorders in CWE can be complicated by the presence of neurocognitive deficits and the lack of validated screening tools or diagnostic criteria in this population. For example, there are few assessment tools for children with intellectual impairment and anxiety (Plevin & Smith, 2019). Careful consideration is required when prescribing for this patient group as anti‐epileptic medications may have psychiatric side effect profiles and psychiatric medications may interact with anti‐epileptic drugs (Plevin & Smith, 2019). As psychiatric comorbidity in CWE increases impairment and early diagnosis of psychiatric problems is key to improving clinical outcomes, functioning and quality of life (Plevin & Smith, 2019), effective collaboration between specialities optimises patient management.

### Common chronic physical disorders

It has been invaluable to have mental health as part of my treatment in the hospital. I am anxious and I know anxiety makes my symptoms worse. I was referred to Mental Health services in the community, but they didn’t understand IBD [inflammatory bowel disease], and they didn’t work out the connection. They thought that I had an eating disorder because I was underweight. I need somebody that understands both and the impact on each other. [13 year old with IBD]

Asthma and type 1 diabetes (T1DM) are amongst the most frequently encountered chronic physical disorders in children’s hospitals. A strong evidence base demonstrates the increased prevalence of psychological disorders in patients with these conditions (Dudeney et al., 2017, Peters & Fritz, 2011, Rechenberg et al., 2017, Winston, 2020). Optimal management relies upon supporting, educating and empowering patients and families to understand the condition, including the importance of adhering to treatment to minimise the risk of acute and long‐term complications. Studies have shown a correlation between psychiatric disorders and lower adherence to treatment with resulting poorer health outcomes (Rechenberg et al., 2017, Winston, 2020, Young et al., 2013). Recognising the prevalence of psychiatric comorbidity in young people with T1DM needs to be integral to their management.

A review by Dudeney et al. (2017) found that the prevalence of anxiety disorders in adolescents under the age of 18 years with asthma is more than three times higher than the general population. The prevalence of depression, ADHD, behavioural disorders and intellectual impairment is also higher in this population (Peters & Fritz, 2011). The prevalence of depression in children aged 3–18 years with T1DM is estimated to be twice that of the general population, whilst the prevalence of anxiety is similarly increased and estimated to be 18.4% (Rechenberg et al., 2017). Studies have also shown an increased prevalence of eating disorders in adolescents with diabetes. For example, a recent review reported a prevalence of 10% in 12–19 year old females with T1DM compared to 4% in controls without diabetes, with even higher rates of subthreshold disorders (Winston, 2020). Comorbid psychiatric disorders affect the management of chronic disease in a number of ways. Whilst emotional triggers are a common precipitant of ‘asthma attacks’, people with asthma can also confuse symptoms of anxiety with asthma, which can lead to a cycle of overuse of short‐acting reliever inhalers (Goodwin et al., 2012). Both anxiety and depression are correlated with a higher asthma symptom burden (Peters & Fritz, 2011). Similarly, in T1DM, psychiatric disorders are associated with poorer disease control, increasing the risk of elevated HbA1c levels and acute and chronic complications (Rechenberg et al., 2017; Winston, 2020; Young et al., 2013).

### Psychiatric emergencies presenting to children’s hospitals

Adolescents with psychiatric emergencies, such as deliberate self‐harm (DSH), severe intoxication and delirium frequently present to medical rather than psychiatric settings, such as emergency departments (ED). A recent UK e‐cohort study reported an increase in ED attendances for self‐harm in 15–19 year olds for both females and males over the past decade, with a corresponding increase in general hospital admissions of females with self‐harm (Marchant et al., 2020). Similarly, in Victoria, Australia, an analysis of presentations to emergency departments in 0–19‐year‐olds from 2008 to 2015 found a disproportionate increase in the number of presentations for psychiatric disorders compared to physical disorders (46% vs. 13%; Hiscock et al., 2018) with self‐harm presentations having increased by 53% to become the most frequent emergency room presentation out of six broad diagnostic groups of psychiatric disorder. Follow up in the community is an important aspect of management, yet there is much variation in service provision and as a recent medical trainee noted, this component is important: *Psychological medicine services help to contain the anxieties of other professionals in the MDT who may not be used to managing distressed patients or necessarily understand the pathways of child and adolescent mental health services in the community [medical trainee, personal communication]*. One UK study reported that 27% of ED attendances for DSH were associated with a subsequent outpatient appointment (Marchant et al., 2020), with studies examining compliance with agreed discharge plans reporting around 66% compliance in a study from Ohio, United States (Sobolewski et al., 2013) and 80% from an Australian study (median age 14 years, majority of presentations for self‐harm; Hopper et al., 2011).

In the acute hospital, especially in intensive care units, delirium is another common psychiatric emergency, which is seen in up to 50% of critically ill children and adolescents (Wilson et al., 2020). Management often involves prescription of psychotropic medication and a thorough understanding of possible underlying somatic conditions, which necessitates appropriate psychiatric input to support acute care, educate staff and families, and plan follow‐up.

### Disorders that commonly present to paediatric services but where psychiatric input is often required

Paediatricians don’t understand mental health and sometimes say things that are inappropriate, and make you feel worse about yourself. Mental health people are not confident with physical stuff and often panic and send you to hospital. Maybe if physical and mental health were more together they could learn from each other and provide what is best for the patient. [14 year old female with an eating disorder]

There should be full collaboration between mental health and physical health services, and they should all be in the same place. I really think it would help to break the stigma about mental health. I think young people would be more open about their mental health, and feel less alone, if it was like any other medical service. It would be more chilled out and more relatable for young people. Stigma is still there, and some young people don’t want to come forward about their mental health because of it. …. When I was seen by the psychological medicine service in the hospital, I found it easier to talk about my mental health because I felt it was taken more seriously. I felt that they looked at the whole picture, not just my behaviour, but everything. In CAMHS [Child and Adolescent Mental Health Services] it felt like it was more routine, less important. [15 year old female with somatic symptom disorder]

The management of some conditions requires both psychiatric and paediatric expertise. These include the management of gender dysphoria, chronic pain syndromes, eating disorders and somatic symptom (somatoform) disorders (see Appendix S1). For these conditions, best clinical care relies upon fully integrated collaborative working and decisions around ‘patient ownership’ that are based on the best interests of the patient rather than historical practice. Adolescents with gender dysphoria have higher rates of depression, autism spectrum disorders (ASDs), suicidality, self‐harm and eating disorders compared to peers (Connolly et al., 2016, Kaltiala‐Heino et al., 2018). Management of gender dysphoria requires thorough psychological assessment as well as input from a range of specialities to provide the medical and surgical treatment that may form part of a patient’s gender transition. The complexity of potential treatments reinforces the value of a fully integrated service. Anxiety and depression are prevalent in paediatric chronic pain syndromes, which are recognised to play an important part in the pain cycle. For example, the prevalence of anxiety disorders in chronic pain has been estimated to exceed 80% (Jastrowski Mano et al., 2019). There have also been reports of children and adolescents with neurodevelopmental disorders, such as ASD, having higher prevalence rates of pain, which similarly highlights the importance of incorporating psychological expertise in management (Whitney & Shapiro, 2019). The management of many chronic pain syndromes as well as the more severe presentations of eating disorders benefit from a multi‐disciplinary team approach to optimise use of pharmacological and non‐pharmacological strategies to manage symptoms and promote normal function.

## A LEGACY OF SEPARATION

Whilst paediatricians commonly work in primary care, community and hospital settings, the majority of child and adolescent psychiatrists are based in separate mental health services that are not part of acute hospital settings. The origins of this separation between physical and mental illness occurred at the beginning of the development of child psychiatry as a specialist area of medical practice in the 1930s.

In the seventeenth century, the philosopher John Locke described the mind of a child as ‘white paper, void of all characters, without any ideas’ (Karafyllis, 2018). This ‘tabula rasa’ kind of thinking contributed to assumptions that children could therefore not experience mental illness. In the nineteenth century, public health and education reforms in the United Kingdom led to improvements in sanitation, campaigns for women’s health and universal education, with some children identified as unable to meet expectations either in learning, behaviour or because of ‘fragility’. Prior to this, children had not been treated separately from adults but were seen as miniature adults, expected to work and be useful within the family and society. Children who deviated from behavioural norms were regarded as deserving of punishment rather than benefitting from medical diagnosis or treatment, and those with intellectual impairments or psychiatric disorders were highly stigmatised. Children as young as five would be managed by general physicians and be admitted to asylums, the forerunners of adult psychiatric wards (Parry‐Jones, 1989).

In the twentieth century, several new strands of thought were beginning to be applied to mental illness in children and adolescents. Psychotherapeutic approaches, which had begun to have a following for adults, were becoming available for children, led by psychologists, such as Anna Freud and Melanie Klein, and paediatricians, such as Donald Winnicott, amongst others. In parallel with child psychotherapy and psychology, the Child Guidance movement started in the United States in the 1930s (Rey et al., 2015). This became a model for working with children with behavioural problems and delinquency. The Child Guidance Centres included social workers, educational psychologists and psychotherapists who worked as a team with the belief that the child must be understood in the context of the family and social environment. Around this time a related move in the United States led a group of paediatricians – who had previously believed in the ‘menace of psychiatry’ – to establish one of the first examples of collaborative care with a paediatric psychiatry unit in the John’s Hopkins ‘Harriet Lane Home for Invalid Children’ (Vicedo & Ilberbaig, 2020).

The interwar years brought an interest in child welfare and an expansion of the treatments for children. After WWII, concerns in the United Kingdom about the effects on children of starvation, deprivation and trauma led to service developments, such as Child Guidance Centres which were primarily based in the community. Becoming a focus for research as well, these centres eventually also housed child psychiatry, as envisioned in the 1946 UK Blacker report. Emphasis on the highly specialist nature of child psychiatry, available to only a few, meant that services were never designed or resourced to meet the true need in the population (Barrett, 2019).

In spite of the good intentions enshrined in the Blacker report, the imposition of psychiatrists into Child Guidance Centres was not always popular. There could be a struggle for dominance in teams, with interdisciplinary rivalry and disagreement about which explanatory model (attachment theory, psychodynamic psychotherapy, family therapy, ecological systems theory, genetic, etc.) was best placed to inform intervention decisions for children. Furthermore, at this time the ‘medical model’ became seen as being opposed to more holistic thinking about the needs of the individual child (Williams & Kerfoot, 2005).

Child psychiatry as an area of specialist medicine was doubly stigmatised in the United Kingdom. Within Child Guidance Clinics and their successors, Child and Adolescent Mental Health Services (CAMHS), psychiatry was stigmatised for medicalising patients, whilst at the same time, it was perceived by mainstream medicine as not being sufficiently medical compared to other specialities. These tensions no doubt affected recruitment and morale in child psychiatry in the United Kingdom and elsewhere. The lack of integrated training pathways, clinical programmes and research between psychiatry, paediatrics and paediatric neurology led to further neglect of the combined medical and psychological needs of some children and adolescents.

The development of the academic discipline of child psychiatry contributed to identifying and quantifying groups amongst the child and adolescent population who had diagnosable psychiatric disorders. Amongst the most notable of research studies was Michael Rutter’s Isle of Wight Study (1970) which laid the foundation of the role of robust epidemiology (Rutter, 1989). Later introductions of disease‐based classification systems for clinical purposes (e.g. The International Classification of Disease) and research (e.g. Diagnostic and Statistical Manual of Mental Disorders) also helped to better identify mental health disorders in children and adolescents.

## NEW MODELS OF INTEGRATION

**FIGURE 2:**
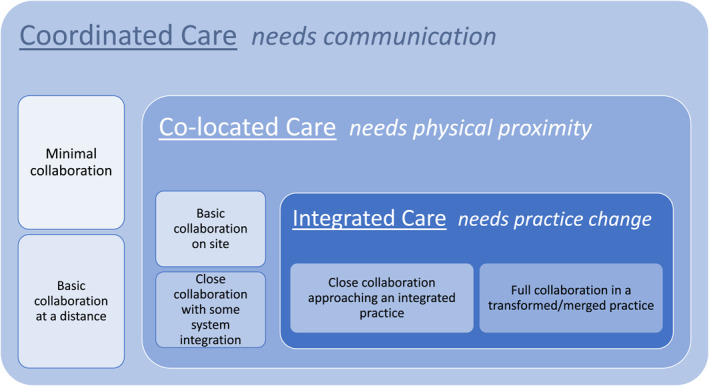
Levels of Collaboration in models of Integrated Care. Adapted from Heath et al. (2013). Deeper blue is intended to signal a higher level of collaboration

This historical legacy has left children’s hospitals in many countries with mental and physical healthcare that continues to be delivered in a fragmented, often separated manner which can compromise access, quality and efficiency of care (Druss & Goldman, 2018). Newer models of care can serve as examples of how services could do much better by being more closely aligned. Figure 2, based on Heath et al. (2013) description of services, highlights the wide variety of collaborative, co‐located and integrated service models that can be adopted. Each service will need to reflect the varied needs of their patients, local availability of resources and the management and political will to integrate psychiatry into the acute children’s hospital environment where the non‐biological aspects of care can otherwise tend to be neglected.

A spectrum of integrated care can be found in various countries including in the United States, where three levels of integration can be considered. The first level involves efforts to promote communication between individual medical and psychiatric providers, who may both need to make an effort to communicate how each other works, as well as their goals of treatment. This approach can be taken to scale, notwithstanding limited availability of child psychiatrists. For example, in the United States, several states have developed publicly‐funded mental health telephone consultations for outpatient paediatricians in an attempt to facilitate access to quality psychiatric care (Dillon‐Naftolin et al., 2017, Wissow et al., 2017). Greater experience of online consultations as a result of the COVID‐19 pandemic is likely to increase the acceptability of these approaches, just as the pandemic has rapidly advanced the implementation of synchronous telehealth consultations in many countries where many of the legal and financial complexities have been rapidly addressed.

The second level more specifically addresses care coordination. Whilst co‐location of psychiatry and primary care improves access, the benefits are not fully reaped in the absence of collaborative care models, such as when a care manager coordinates care between primary care clinicians and specialists. A recent review of paediatric integrated and collaborative care models found that increased access to psychiatric treatment can improve outcomes for medically ill children and adolescents (Burkhart et al., 2020).

Beyond care coordination, a third step to integration is when paediatric and child psychiatry systems more seamlessly promote coordinated care, including through shared access to medical records. Different services are developing new models of embedding mental health specialists in speciality clinics. This includes specialist paediatric eating disorder services where access to multidisciplinary care is increasingly the norm or having psychologists based within epilepsy clinics as services become increasingly focused on outpatients (Hughes et al., 2014, Plevin & Smith, 2019). As with adult inpatient hospital services (Sharpe et al., 2020), paediatric inpatient services are also exploring proactive provision, such as the Children’s Psychological Medicine Service in Oxford University Hospitals, where integrated care ensures psychiatrists and psychologists attend the daily rounds of all new paediatric admissions to proactively identify those children and adolescents who would benefit from an integrated approach.

Yet the dead hand of history often impedes change, which can take considerable goodwill, time, effort and investment to shift (Druss & Goldman, 2018). Change requires a step‐wise approach towards integration, with an appreciation that trust needs to be established between the people running services. Addressing organisational challenges, key financing questions and differing service values takes time (Druss & Goldman, 2018). Physical co‐location can be an important early step to facilitate closer professional collaboration and the development of a shared vision (Peek & National Integration Academy Council, 2013). Table 1 highlights some professional opportunities that psychiatrists can view as helpful steps towards closer collaboration.

**TABLE 1:** Opportunities that can enhance collaboration in daily practice

Opportunity	Possible solutions/responses
Individual complex cases where paediatric colleagues have requested psychiatric input	Psychiatry input: Technical expertise, knowledge of complex systems (child and family, team challenges) and treatment implications.
Unexplained variation in outcomes, institutional differences, including undue reliance on inpatient care	Development of joint clinical pathways and protocols outlining the roles and responsibilities of medical and psychiatric leads.
Increased communication with a medical service that treats a recognised problem or has many patients with similar difficulties (such as in a diabetes service)	There can be advantages of piloting a service in one specific area in order to address clinical and service delivery challenges, measure impacts and use this to explore possible solutions for other services.
Individual paediatricians or groups of health care staff who have identified that they are finding certain cases difficult or are avoiding exploring certain aspects of care, including psychiatric components	Training to enhance individual psychological skills, appreciation of developmental and intergenerational perspectives and confidence at balancing the bio‐psycho‐social components of disorders. This is likely to lead to improve individual staff member’s wellbeing, enhance team function and improve patient outcomes.
Increasing referrals for psychiatric assessment and care	Review the efficiency of referral processes, pathways and feedback.
Leadership interested in wider service changes	Support for change can augment the courage and energy required to overcome system inertia. This can be aided by outcome data, including cost‐effectiveness of integrated care models.

In the United States, a number of important innovations have been developed around integrated psychiatric and paediatric training that can help support more integrated models of clinical care. For example, in the mid‐1980s a combined training programme was set up in a few centres to bring paediatrics, psychiatry and child psychiatry together in the formation of Triple Board training (Gleason & Sexson, 2017). More recently, a ‘Pediatric portal’ programme was established to encourage board certified paediatricians to supplement their skills through additional formal training in psychiatry, as well as discussions about the common core competencies required within each speciality (Gleason & Sexson, 2017, Shaw et al., 2019). Amongst other outcomes, these approaches are expected to build trust between psychiatrists and paediatricians.

Child and adolescent psychiatrists working in hospital systems need to ensure they have the expertise to encompass the complexity, treatment implications and medical systems being navigated – not just for a specific patient, but by the ward, team or even hospital. Their professional competencies need to contribute to the diagnosis, formulation and treatment plans developed by utilising core skills that bring together biological, psychodynamic, pharmacological, developmental, family and systems perspectives into the consultation. The growing awareness of the psychological landscape of the child and adolescent being treated, can be more easily acknowledged and explored when medical, nursing and allied health staff have strong appreciation of the value of providing psychological assessment and interventions within more integrated models of care in children’s hospitals. In addition, the presence of mental health professionals within services might enhance skill building for all staff, including through clinical discussions and supervision, improving everyone’s capacity to acknowledge and address the psychological needs of children and their families, as well as the clinical teams that support them.

Observable outcomes might be that difficult emotional situations become easier to manage within the service, or that with the presence of a psychiatrist, enquiring about the broader individual or family context is not avoided. Innovations using telehealth consultations, or enhanced peer support groups are likely to contribute but need evidence to support greater adoption by services. Compelling reasons to integrate psychiatry into primary care have been successfully made by evidencing the burden of mental disorders and addressing the treatment gap of needed versus available psychiatric services (Funk & Ivbijaro, 2008).

## A CALL FOR ACTION

Increasing numbers of children and adolescents with life‐limiting, complex chronic health conditions and disabilities are presenting to medical services with complex psychological needs. Psychiatric comorbidity affects adherence to medical treatment, health outcomes, quality of life and global functioning in the foundational years of human development. Yet contemporary clinical contexts, characterised by increasing sub‐specialisation and high levels of technical competence, risk reinforcing the legacy of distinct physical and mental health services, eroding opportunities for more integrated approaches for patient‐centred and family focussed healthcare delivery. Improved experiences of care, better quality health of the population and reduced healthcare costs for individuals are compelling reasons for more integrated services (Kullgren et al., 2020).

As the majority of major lifelong mental illnesses start in the adolescent years when patterns of self‐care and care‐seeking become established, approaches to engaging young people with chronic health conditions around both their physical and emotional wellbeing are important. Adolescence can be especially complex for this cohort whose interpersonal relationships with family, schools and peers can be as disrupted by the complexity of their disorders and the healthcare systems they must negotiate (Patterson & Vakili, 2014, Rickerby & Roesler, 2015, Sawyer et al., 2007). For those with uncertain prognoses or life‐limiting conditions, the additional challenges of managing difficult symptoms (e.g. pain, fatigue) and repeated hospital appointments or admissions can exact a heavy burden on their emotional wellbeing, friendships and family life (Barker et al., 2019).

Despite increasing numbers of young people growing up with chronic and life‐limiting conditions (Barker et al., 2019), there is continued inertia within the health system around approaches to better prevent, diagnose and treat comorbid psychiatric illness. Embedded within our legacy of separated physical and mental health services, this inertia is supported by separate institutions, reflected in independent training and reinforced by distinct funding schemes. Child and adolescent health and wellbeing would be greatly supported by research in and development of creative service models that enhance integration. The COVID‐19 pandemic has contributed to a mental health crisis in young people and resulted in a groundswell of interest in child and adolescent emotional wellbeing. Now is the time for the field to grasp the opportunity of responding, not by simply doing more of the same (as we are not meeting the needs of paediatric patients and their families) but by considering opportunities for doing things differently.

Finally, the opportunities to be gained extend beyond some of these examples in high‐resource settings. Child psychiatry is even more poorly accessible in resource‐poor countries where paediatric care remains focussed on acute care, where mental health literacy is particularly low and mental illness remains highly stigmatised (Sawyer et al., 2019). These countries have even more to gain than high income countries in developing cost‐effective, integrated models of care which can address both physical and mental health and wellbeing.

## CONFLICT OF INTEREST

The authors have declared that they have no competing or potential conflicts of interest.

## ETHICS STATEMENT

Formal ethical review was not required for this clinical review which was based on previously published studies.

## AUTHOR CONTRIBUTIONS

All authors worked on conceptualisation. Mina Fazel wrote the first draft. Alice Townsend conducted, summarised and wrote the literature review. Harriet Stewart wrote the historical review. Isabel Paz conducted interviews of the adolescents. All authors reviewed and helped refine the manuscript at all stages of the work.

## Supporting information

Supporting Information S1

Supporting Information S2

## Data Availability

Data sharing not applicable to this article as no datasets were generated or analysed during the current study.
