# Supplementary material for: Integrated care to address child and adolescent health in the 21st century: A clinical review
Source: JCPP Adv. 2021 Oct 23;1(4):e12045. doi: 10.1002/jcv2.12045 (PMC10242873; doi:10.1002/jcv2.12045)
Supplement: Supplementary file 1 — Supporting Information S1 [file JCV2-1-e12045-s001.docx]

## Appendix S1: Summary of findings from reviews of psychiatric comorbidities in common conditions

| **Illness** | **Epidemiology of psychiatric comorbidity** | **Example of the effects of psychiatric comorbidity** |
| --- | --- | --- |
| **Cancer**  *Kurtz 2011, Lanier 2017, Shah 2015, Temming 2010* | - Leukaemia survivors aged 12-17 years have 1.5x risk of depression/anxiety symptoms compared to siblings (Temming and Jenney, 2010). - >50% of those treated for childhood cancer (aged 0-21 years) are at risk of developing neurocognitive deficits (Kurtz and Abrams, 2011). - CNS tumour survivors (diagnosis at age 0-18 years) are at increased risk of depression, anxiety, schizophrenia and related psychoses, and behavioural problems compared to general population (Shah *et al.*, 2015). - Following posterior fossa tumour resection aged 3-22 years approximately 25% develop syndrome of behavioural changes, mutism and ataxia (Lanier and Abrams, 2017). - Severe late effects increase the risk of depression (Kurtz and Abrams, 2011). - Cranial radiation is a major risk factor for neurocognitive deficits (Kurtz and Abrams, 2011). | - Anticipatory anxiety and separation anxiety are associated with frequent investigations and treatment and can interfere with medical care (Kurtz and Abrams, 2011). |
| **Epilepsy**  *Plevin 2019, Plioplys 2003, Reilly 2011, Salpekar 2015, Verrotti 2014* | - 37-77% have psychiatric comorbidity (Verrotti *et al.*, 2014). - 30-35% (0-18 years) have anxiety disorders (Plevin and Smith, 2019, Reilly *et al.*, 2011). - 13-37% (0-18 years) have depression (Plevin and Smith, 2019, Reilly *et al.*, 2011). - ADHD is the most frequent psychiatric comorbidity in children aged 4-12 years (Verrotti *et al.*, 2014). - Depression is the most frequent psychiatric comorbidity in adolescents aged 13-17 years (Verrotti *et al.*, 2014). - Poorly controlled epilepsy is associated with 10x risk of depression (Salpekar *et al.*, 2015). - If epilepsy is adequately controlled, the risk of depression is still 2-5x higher (Salpekar *et al.*, 2015). | - Stress is a common self-reported precipitant of seizures (Verrotti *et al.*, 2014). |
| **Traumatic Brain Injuries (TBIs)**  *Laliberte Durish 2018, Max 2014, Trenchard 2013* | - Novel psychiatric disorders (including personality change, secondary attention-deficit/hyperactivity disorder, disruptive behavior disorders and internalizing disorders) are present after 24 months in 54-63% post-severe TBI, 10-21% post-mild to moderate TBI (Max, 2014). - 33-50% following mild to severe TBI have depression (ages 5-18 years) (Laliberte Durish *et al.*, 2018). - Brain lesions, pain, more severe injury, older age and low socioeconomic status are associated with increased risk of psychiatric comorbidity (Laliberte Durish *et al.*, 2018, Trenchard *et al.*, 2013). | - Depressive symptoms are associated with increased risk of persistent pain and poorer school functioning post-TBI (Laliberte Durish *et al.*, 2018). |
| **Asthma**  *Brady 2017, Dudeney 2017, Goodwin 2012, Peters 2011* | - Increased rates of depression, attention-deficit/hyperactivity disorder (ADHD), behavioral disorders, and learning disabilities at ages 0-17 years (Brady *et al.*, 2017, Peters and Fritz, 2011). - 49% (9-17 years) have anxiety disorders (Peters and Fritz, 2011). - Prevalence of ≥ 1 anxiety or depressive disorder (11-17 years) is nearly 2x greater than controls (Peters and Fritz, 2011). - Severity of asthma symptoms correlates with likelihood of depression, learning disabilities, ADHD and other behavioural disorders (Peters and Fritz, 2011). | - Comorbid anxiety and depression are associated with increased risk of smoking (Peters and Fritz, 2011). |
| **Diabetes**  *Buchberger 2016, Jaser 2010, Kongkaew 2014, Racicka 2015, Rechenberg 2017, Toni 2017, Winston 2020, Young 2013* | - 11-28% of children and adolescents (primarily 8-18 years) with Type 1 diabetes have depression and 13-21% have anxiety (Rechenberg *et al.*, 2017). - Disordered eating behaviour and eating disorders are more common in adolescents with Type 1 diabetes compared with peers (Young *et al.*, 2013). - Binge eating disorder is the most common eating disorder in adolescents with Type 2 diabetes (Winston, 2020). - Females are at higher risk of anxiety, fear of hypoglycaemia and eating disorders (Rechenberg *et al.*, 2017). | - Depression, anxiety and eating disorders are associated with poorer glycaemic control and therefore increased risk of acute and chronic complications (Kongkaew *et al.*, 2014, Rechenberg *et al.*, 2017, Toni *et al.*, 2017, Winston, 2020, Young *et al.*, 2013). |
| **Inflammatory Bowel Disease (IBD)**  *Avila 2019, Keethy 2014, Mackner 2013, Ross 2011, Sajadinejad 2012* | - From 11- 25% have depression in those aged under 18 years (Mackner *et al.*, 2013). - Increased risk of psychiatric comorbidity with pain, sleep disturbance, IBD-related inflammation, diminished control over life and future, embarrassment, perception of being different (Keethy *et al.*, 2014). | - Psychiatric comorbidity is associated with poorer treatment adherence (Keethy *et al.*, 2014). |
| **Chronic pain (CP)**  *Jastrowski 2019, Soltani 2019, Vinall 2016* | - 44% (mean age 13 years) have psychiatric comorbidity (Vinall *et al.*, 2016). - Higher lifetime rates of anxiety (21%) and depressive (25%) disorders (age 12-32 years) (Vinall *et al.*, 2016). - 2x rate of eating disorders (Vinall *et al.*, 2016). - Increased risk of psychiatric comorbidity associated with cognitive biases, sleep disturbances, emotional dysregulation, behavioural inactivation, parental psychiatric disorders, pain, genetic factors, parenting issues (Soltani *et al.*, 2019)   . | - Premorbid depression may increase the likelihood of acute pain evolving into chronic pain (Soltani *et al.*, 2019). |
| **Gender dysphoria, Transgender (TGN)**  *Connolly 2016, Guss 2015, Kaltiala-Heino 2018* | - 40-45% psychiatric disorders in adolescents referred to gender identity clinic (GIC) with 6-20% having autism spectrum disorders (Kaltiala-Heino *et al.*, 2018); - 257-64% adolescents presenting to GIC have depression (Connolly *et al.*, 2016). - 16% transgender students aged <26 years have eating disorders (Connolly *et al.*, 2016). - In community samples of adolescents, depression 4-6x more common and self-harm/suicidal behaviour 3-4x more common compared to cisgender (Kaltiala-Heino *et al.*, 2018). - Higher risk of depressive symptoms associated with onset of gender nonconformity before age 11 years (Guss *et al.*, 2015). | - Psychiatric comorbidity increases the complexity of decision-making regarding treatments, especially those with significant or irreversible effects (Guss *et al.*, 2015). |
| **Skin Conditions (acne, eczema, dermatitis, psoriasis)**  *Dunn 2011, Gieler 2015, Gonzalez 2016, Habeshian 2020, Kage 2021, Natsuaki 2021, Rabin 2012, Revol 2015* | - Significant increase in risk of depression, anxiety and suicidal ideation with acne (Dunn LK, 2011, Gieler *et al.*, 2015, Natsuaki and Yates, 2021). - Increased risk of ADHD (children) and suicidal ideation (adolescents) with atopic dermatitis (Kage *et al.*, 2020). - 25-47% higher risk of any psychiatric disorder, 23-62% higher risk of depression and 32-250% higher risk of anxiety with psoriasis (Rabin *et al.*, 2012). - Increased risk of psychiatric comorbidity with scarring and severity of acne (Natsuaki and Yates, 2021, Revol *et al.*, 2015). | - Psychiatric comorbidity exacerbates difficulties with treatment adherence (Gieler *et al.*, 2015, Habeshian and Cohen, 2020). |
| **Obesity**  *Byrne 2015, Harriger 2012, Kalarchian 2012, Maloney 2011, Matheson 2018,*  *Puder 2010, Rao 2020, Sutaria 2019,Vander Wal 2011* | - Loss of control of eating behaviour is prevalent (Puder and Munsch, 2010). - Increased risk of depression (aged <18 years) (Sutaria *et al.*, 2019). - Presence of another chronic illness, familial stress and female sex associated with increased risk of psychiatric comorbidity (Kalarchian and Marcus, 2012, Puder and Munsch, 2010, Sutaria *et al.*, 2019). | - Depression is associated with higher rates of treatment drop-out and with weight gain (Matheson and Eichen, 2018, Vander Wal and Mitchell, 2011). |

Explanatory note:

We compiled a list of common chronic illnesses seen in paediatric medicine using relevant literature (Wiljaars LPMM, 2016, Traves D, 2014) then conducted a literature search (using Ovid MEDLINE Epub ahead of Print, IN-Process & Other Non-Indexed Citations, Ovid MEDLINE Daily and Ovid MEDLINE) to identify relevant review articles published since 2010 on psychiatric comorbidities in children and adolescents with these illnesses. We included illnesses in this table when we were able to identify at least three relevant reviews.

Avila, J. T., Park, K. T., & Golden, N. H. (2019). Eating disorders in adolescents with chronic gastrointestinal and endocrine diseases.  *Lancet Child & Adolesc Health*, *3*(3), 181-189.

Brady, A. M., Deighton, J. & Stansfeld, S. (2017). Psychiatric outcomes associated with chronic illness in adolescence: A systematic review. *J Adolesc* 59, 112-123.

Buchberger, Barbara, Hendrik Huppertz, Laura Krabbe, Beate Lux, Jessica T. Mattivi, and Aris Siafarikas. "Symptoms of depression and anxiety in youth with type 1 diabetes: A systematic review and meta-analysis." *Psychoneuroendocrinology* 70 (2016): 70-84.

Byrne, M. L., O’Brien-Simpson, N. M., Mitchell, S. A., & Allen, N. B. (2015). Adolescent-onset depression: are obesity and inflammation developmental mechanisms or outcomes?. *Child Psychiatry & Human Development*, *46*(6), 839-850.

Connolly, M. D., Zervos, M. J., Barone, C. J., 2nd, Johnson, C. C. & Joseph, C. L. (2016). The Mental Health of Transgender Youth: Advances in Understanding. *J Adolesc Health* 59, 489-495.

Dudeney, J., Sharpe, L., Jaffe, A., Jones, E. B. & Hunt, C. (2017). Anxiety in youth with asthma: A meta-analysis. *Pediatic Pulmonology* 52, 1121-1129.

Dunn LK, O. N. J., Feldman SR (2011). Acne in adolescents: quality of life, self-esteem, mood and psychological disorders. *Dermatol Online J* 17.

Gieler, U., Gieler, T. & Kupfer, J. P. (2015). Acne and quality of life - impact and management. *J Eur Acad Dermatol Venereol* 29 Suppl 4, 12-4.

Gonzalez, J., Cunningham, K., Perlmutter, J., & Gottlieb, A. (2016). Systematic review of health-related quality of life in adolescents with psoriasis. *Dermatology*, *232*(5), 541-549.

Goodwin, R. D., Bandiera, F. C., Steinberg, D., Ortega, A. N. & Feldman, J. M. (2012). Asthma and mental health among youth: etiology, current knowledge and future directions. *Expert Review of Respiratory Medicine* 6, 397-406.

Guss, C., Shumer, D. & Katz-Wise, S. L. (2015). Transgender and gender nonconforming adolescent care: psychosocial and medical considerations. *Curr Opin Pediatr* 27, 421-6.

Habeshian, K. A. & Cohen, B. A. (2020). Current Issues in the Treatment of Acne Vulgaris. *Pediatrics* 145, S225-S230.

Harriger, J. A., & Thompson, J. K. (2012). Psychological consequences of obesity: weight bias and body image in overweight and obese youth. *Int Rev Psychiatry*, *24*(3), 247-253.

Jaser, S. S. (2010). Psychological problems in adolescents with diabetes. *Adolescent medicine: state of the art reviews*, *21*(1), 138.

Jastrowski Mano, K. E., O'Bryan, E. M., Gibler, R. C. & Beckmann, E. (2019). The Co-occurrence of Pediatric Chronic Pain and Anxiety: A Theoretical Review of a Developmentally Informed Shared Vulnerability Model. *Clinical Journal of Pain* 35, 989-1002.

Kage, P., Simon, J. C. & Treudler, R. (2020). Atopic dermatitis and psychosocial comorbidities. *J Dtsch Dermatol Ges* 18, 93-102.

Kalarchian, M. A. & Marcus, M. D. (2012). Psychiatric comorbidity of childhood obesity. *Int Rev Psychiatry* 24, 241-6.

Kaltiala-Heino, R., Bergman, H., Tyolajarvi, M. & Frisen, L. (2018). Gender dysphoria in adolescence: current perspectives. *Adolesc Health Med Ther* 9, 31-41.

Keethy, D., Mrakotsky, C. & Szigethy, E. (2014). Pediatric inflammatory bowel disease and depression: treatment implications. *Curr Opin Pediatr* 26, 561-7.

Kongkaew, C., Jampachaisri, K., Chaturongkul, C. A. & Scholfield, C. N. (2014). Depression and adherence to treatment in diabetic children and adolescents: a systematic review and meta-analysis of observational studies. *Eur J Pediatr* 173, 203-12.

Kurtz, B. P. & Abrams, A. N. (2011). Psychiatric aspects of pediatric cancer. *Pediatr Clin North Am* 58, 1003-23, xii.

Laliberte Durish, C., Pereverseff, R. S. & Yeates, K. O. (2018). Depression and Depressive Symptoms in Pediatric Traumatic Brain Injury: A Scoping Review. *J Head Trauma Rehabil* 33, E18-E30.

Lanier, J. C. & Abrams, A. N. (2017). Posterior fossa syndrome: Review of the behavioral and emotional aspects in pediatric cancer patients. *Cancer* 123, 551-559.

Mackner, L. M., Greenley, R. N., Szigethy, E., Herzer, M., Deer, K. & Hommel, K. A. (2013). Psychosocial issues in pediatric inflammatory bowel disease: report of the North American Society for Pediatric Gastroenterology, Hepatology, and Nutrition. *J Pediatr Gastroenterol Nutr* 56, 449-58.

Maloney, A. E. (2011). Pediatric obesity: a review for the child psychiatrist. *Pediatric Clinics*, *58*(4), 955-972

Matheson, B. E. & Eichen, D. M. (2018). A Review of Childhood Behavioral Problems and Disorders in the Development of Obesity: Attention Deficit/Hyperactivity Disorder, Autism Spectrum Disorder, and Beyond. *Curr Obes Rep* 7, 19-26.

Max, J. E. (2014). Neuropsychiatry of pediatric traumatic brain injury. *Psychiatr Clin North Am* 37, 125-40.

Natsuaki, M. N. & Yates, T. M. (2021). Adolescent Acne and Disparities in Mental Health. *Child Development Perspectives* 15, 37-43.

Peters, T. E. & Fritz, G. K. (2011). Psychological considerations of the child with asthma. *Pediatr Clin North Am* 58, 921-35, xi.

Plioplys, S. (2003). Depression in children and adolescents with epilepsy. *Epilepsy & Behavior*, *4*, 39-45.

Plevin, D. & Smith, N. (2019). Assessment and Management of Depression and Anxiety in Children and Adolescents with Epilepsy. *Behav Neurol* 2019, 2571368.

Puder, J. J. & Munsch, S. (2010). Psychological correlates of childhood obesity. *Int J Obes (Lond)* 34 Suppl 2, S37-43.

Rabin, F., Bhuiyan, S., Islam, T., Haque, M. & Islam, M. (2012). Psychiatric and psychological comorbidities in patients with psoriasis-a review. *Mymensingh medical journal: MMJ* 21, 780-786.

Racicka, E., & Bryńska, A. (2015). Eating disorders in children and adolescents with type 1 and type 2 diabetes: prevalence, risk factors, warning signs. *Psychiatr Pol*, *49*(5), 1017-1024

Rao, W. W., Zong, Q. Q., Zhang, J. W., An, F. R., Jackson, T., Ungvari, G. S., ... & Xiang, Y. T. (2020). Obesity increases the risk of depression in children and adolescents: Results from a systematic review and meta-analysis. *Journal of affective disorders*, *267*, 78-85.

Rechenberg, K., Whittemore, R. & Grey, M. (2017). Anxiety in Youth With Type 1 Diabetes. *J Pediatr Nurs* 32, 64-71.

Reilly, C., Agnew, R. & Neville, B. G. (2011). Depression and anxiety in childhood epilepsy: a review. *Seizure* 20, 589-97.

Revol, O., Milliez, N. & Gerard, D. (2015). Psychological impact of acne on 21st-century adolescents: decoding for better care. *Br J Dermatol* 172 Suppl 1, 52-8.

Ross, S. C., Strachan, J., Russell, R. K., & Wilson, S. L. (2011). Psychosocial functioning and health-related quality of life in paediatric inflammatory bowel disease. *Journal of pediatric gastroenterology and nutrition*, *53*(5), 480-488.

Sajadinejad, M.S., et al., *Psychological issues in inflammatory bowel disease: an overview.* Gastroenterol Res Pract, 2012. 2012: p. 10650

Salpekar, J. A., Mishra, G. & Hauptman, A. J. (2015). Key issues in addressing the comorbidity of depression and pediatric epilepsy. *Epilepsy Behav* 46, 12-8.

Shah, S. S., Dellarole, A., Peterson, E. C., Bregy, A., Komotar, R., Harvey, P. D. & Elhammady, M. S. (2015). Long-term psychiatric outcomes in pediatric brain tumor survivors. *Childs Nerv Syst* 31, 653-63.

Soltani, S., Kopala-Sibley, D. C. & Noel, M. (2019). The Co-occurrence of Pediatric Chronic Pain and Depression: A Narrative Review and Conceptualization of Mutual Maintenance. *Clin J Pain* 35, 633-643.

Sutaria, S., Devakumar, D., Yasuda, S. S., Das, S. & Saxena, S. (2019). Is obesity associated with depression in children? Systematic review and meta-analysis. *Arch Dis Child* 104, 64-74.

Temming, P. & Jenney, M. E. (2010). The neurodevelopmental sequelae of childhood leukaemia and its treatment. *Arch Dis Child* 95, 936-40.

Toni, G., Berioli, M. G., Cerquiglini, L., Ceccarini, G., Grohmann, U., Principi, N. & Esposito, S. (2017). Eating Disorders and Disordered Eating Symptoms in Adolescents with Type 1 Diabetes. *Nutrients* 9.

Trenchard, S. O., Rust, S. & Bunton, P. (2013). A systematic review of psychosocial outcomes within 2 years of paediatric traumatic brain injury in a school-aged population. *Brain Inj* 27, 1217-37.

Vander Wal, J. S. & Mitchell, E. R. (2011). Psychological complications of pediatric obesity. *Pediatr Clin North Am* 58, 1393-401, x.

Verrotti, A., Carrozzino, D., Milioni, M., Minna, M. & Fulcheri, M. (2014). Epilepsy and its main psychiatric comorbidities in adults and children. *J Neurol Sci* 343, 23-9.

Vinall, J., Pavlova, M., Asmundson, G. J., Rasic, N. & Noel, M. (2016). Mental Health Comorbidities in Pediatric Chronic Pain: A Narrative Review of Epidemiology, Models, Neurobiological Mechanisms and Treatment. *Children (Basel)* 3.

Wijlaars, Linda PMM, Ruth Gilbert, and Pia Hardelid. "Chronic conditions in children and young people: learning from administrative data." *Arch Dis Child* 101.10 (2016): 881-885.

Winston, A. P. (2020). Eating Disorders and Diabetes. *Curr Diab Rep* 20, 32.

Young, V., Eiser, C., Johnson, B., Brierley, S., Epton, T., Elliott, J. & Heller, S. (2013). Eating problems in adolescents with Type 1 diabetes: a systematic review with meta-analysis. *Diabet Med* 30, 189-98.
