# Supplementary material for: Integrated care to address child and adolescent health in the 21st century: A clinical review
Source: JCPP Adv. 2021 Oct 23;1(4):e12045. doi: 10.1002/jcv2.12045 (PMC10242873; doi:10.1002/jcv2.12045)
Supplement: Supplementary file 2 — Supporting Information S2 [file JCV2-1-e12045-s002.docx]

Appendix S2: A parent’s experience

For years we went to the GP and said our son has mental health issues, and he said “well good luck with that”. I said I was willing to pay for it privately, but there was nothing available, nothing at all. It was horrible. He was misdiagnosed with epilepsy at the age of 10. This occurred after he was having different types of (what appeared to be) seizures over a period of a year. His epilepsy diagnosis and subsequent 5 years of medication was the medical default way of treating what he was experiencing. What followed was years and years of more medication, induced comas and more 'seizures' - it all got progressively worse. Our sweet son turned into a walking zombie. We felt that he would never live an independent life and worse yet, it seemed that he wasn't able to experience joy or look forward. It took a mental health professional to look at his situation differently. That changed everything.

A defining moment that still brings tears to my eyes is when I recall the meeting that we had with our neurologist and psychiatrist. This was the meeting where as a team, the neurologist and psychiatrist told us that they were 95% sure that our son did not have epilepsy. That was a very scary (and also a very hopeful) time for us. Before we left the meeting the doctor locked eyes with our son and said 'on behalf of the medical profession, I apologise'.

That statement was a turning point for our whole family. It took courage to say it and it helped us move on to the next step of getting care. We felt that we could do that because the psychiatrist was part of that process and that conversation. We were turned over to their care, quite literally, on that day with the neurologist in the room.

The miracle for us was when we went to the hospital and someone recognised the need for mental health support. We were in crisis and needed the help there and then. Before the crisis I tried Tai-chi and horse therapy, I just wanted for my son to talk to somebody. There is no preventative care in mental health, and is difficult to get support even if you are in crisis. It is as if people are afraid to acknowledge the need for mental health services, because if they do, someone will have to provide those services. More recently it feels like people are talking more about mental health, even in the Royal family.

Now we feel that our son’s future is bright and he is looking forward.
